# An unreported innervation of the coracobrachialis longus by the radial nerve: a potential pitfall for clinicians

**DOI:** 10.1007/s00276-023-03166-z

**Published:** 2023-05-17

**Authors:** Krystian Maślanka, Nicol Zielinska, Krzysztof Koptas, Łukasz Olewnik, Piotr Łabętowicz

**Affiliations:** Department of Anatomical Dissection and Donation, Medical University of Lodz, Lodz, Poland

**Keywords:** Radial nerve, Additional branch, Coracobrachialis longus, Coracobrachialis, Brachial plexus, Undescribed innervation, Shoulder surgery

## Abstract

**Purpose:**

The aim of the presented case is to describe an unprecedented innervation of the coracobrachialis longus muscle by the radial nerve.

**Methods:**

An 82-year-old body donor at death was subjected to a routine anatomical dissection for teaching and research purposes at the Department of Anatomical Dissection and Donation in Lodz, Poland.

**Results:**

We have found an additional branch of the radial nerve, which departed from it just below its beginning. Its initial section ran alongside the radial nerve in the axilla, then headed medially accompanying superior ulnar collateral artery. Then, it reaches the coracobrachialis longus muscle and is the only one to innervate it.

**Conclusions:**

The brachial plexus (BP) is very variable and well understood. Nevertheless, we must remember that there may still be variations in its structure, which may involve problems at every stage of diagnosis and treatment of diseases associated with its structures. Their knowledge is extremely important.

## Introduction

Radial nerve (RN) is the largest terminal branch of the brachial plexus. It is an extension of posterior cord that provides motor, sensory and sympathetic fibers originating from the C5–C8 and T1 roots. Initially, it runs behind the axillary artery and next behind the brachial artery in the arm. It goes through the triangular interval, winds around the medial side of the humerus and then enters the triceps muscle between lateral and medial heads, simultaneously innervating it. At this point RN, gives two sensory branches, posterior and lateral inferior cutaneous nerve of the arm [20]. Then, it runs into the spiral groove and reaches the anterior compartment by piercing the intermuscular septum. At the elbow level, RN gives branches to the anconeus, extensor carpi radialis longus and brachioradialis [1].

In the forearm, RN divides into deep and superficial branches, which are solely sensory. Initially, the superficial part lies under the brachioradialis muscle where it runs laterally to the radial artery. When RN reaches the level of brachioradialis tendon, is located superficially, and divides into two cutaneous branches which innervate dorsal skin of the hand and the skin that covers the proximal part of the lateral three and a half fingers. The posterior part runs between heads of the supinator muscle, reach the forearm and innervates hand and forearm extensors and abductor pollicis longus [10].

Morphological variations of the radial nerve occur frequently, but a variation observed in its distal is highly common [5, 15, 22]. Claassen et al. [8] in their study showed that in the proximal part of the arm, the radial nerve was the least variable nerve in that part of the arm. Moreover, within the arm, RN very rarely gives additional nerve fibers to muscles that are not standardly innervated by it [19].

The radial nerve is a peripheral nerve of upper extremities that gets injured most frequently. Most of these cases include humerus fractures and compression. The main reason for this is its unfavorable anatomical position [21]. Holstein–Lewis fracture, contributing to high neurovascular incidence, is a characteristic injury in this group of injuries [12].

Additional radial nerve branches within the arm are observed extremely rarely. This report describes case of innervation of the coracobrachialis longus (CBL). To our knowledge, it was not previously described.

## Case report

An 82-year-old deceased woman, donor at death was subjected to a routine anatomical dissection for teaching and research purposes at the Department of Anatomical Dissection and Donation in Lodz. The upper limb and chest area were subjected to a standard anatomical dissection and fixed in 10% formalin solution [14]. The dissection began with the removal of skin, fascia and fat tissue from the lateral part of the chest, shoulder and proximal medial side of the arm. Then, the sternocleidomastoid and omohyoid muscle were cut and pushed away. The exposed clavicle was excised. The next step included visualization of the brachial plexus and its branches, superior ulnar collateral artery, coracobrachialis longus and biceps brachii muscle (BB).

During research on the brachial plexus, new branching of the radial nerve was observed. It departed from RN just 65 mm below its beginning. Its initial section ran alongside the radial nerve in the axilla. Then, it headed medially alongside the superior ulnar collateral artery. Throughout this length, we did not notice any specific anatomical correlation. 13 mm before the place where it pierces the coracobrachialis longus (CL), this new recognized part of BP branched into two parts, i.e., superior and inferior terminal branches. The first one reached the coracobrachialis longus in 173.15 mm of its length, while the other one in 199.35 mm. Superior ulnar collateral arteries penetrated the CL below both of these branches. The length of the branch from its beginning to its branching was 117.2 mm, the superior terminal branch was 13.68 mm long, while the inferior terminal branch was 32.45 mm. The diameter of 0.6 mm was measured along its entire length. CL was innervated only by the radial nerve.

These structures were carefully dissected to minimize errors during measurement, for which we used an electronic caliper (Mitutoyo Corporation, Kawasaki-shi, Kanagawa, Japan). Each measurement was repeated twice with an accuracy of up to 0.1 mm. Table 1 shows morphometric measurements of the presented case.

**Table 1:** Morphometric measurements of particular part of the observed nerve

Length	
From the radial nerve to terminal branching	117.2 mm
Superior terminal branch	13.68 mm
Inferior terminal branch	32.45 mm
Diameter	
Along the entire length	0.6 mm
Coracobrachialis longus puncture points, relative to coracoid process	
Superior terminal branch	173.15 mm
Inferior terminal branch	199.35 mm

## Discussion

Variations of the brachial plexus may appear due to unusual formation in the development of cords, trunks and particular divisions. Mesenchyme begins its differentiation in the fourth week of fetal life. During this period, continuity of BP components become visible. In week 7, the plexus has already reached the first rib level and resembles a three-line structure. Its development begins on days 34–36 of gestation. Median, radial and ulnar nerves reach the palm level on days 39–40. On days 46–48, BP structure is similar to those in adults. Anatomical variations of the brachial plexus can be explained after proper understanding of its normal embryological development [28].

As mentioned above, there are not many variations regarding the radial nerve within the arm and axilla. Claassen et al. [8] conducted an analysis on 167 arms and noted variations only in the one of them (0.6%). The radial nerve in that case appeared to be thinner than it is normally at its beginning. RN and the ulnar and axillary nerve are least variable in their study. Musculocutaneous (MCN) and median nerves were characterized by significantly greater variability than other nerves [8].

Variations of the radial nerve relate primarily to connections with other nerves originating in the brachial plexus and are relatively rare. In the available literature there are few reports concerning such connections of the radial nerve [6]. Konstantinos Natsis et al. [16] in 2018 published three studies in which they confirmed that connections between the radial and ulnar nerves may occur relatively more frequent than it was reported in previous studies. This represented 2.6% of the total 266 detected upper limbs from 133 cadavers [16]. Candan et al. [6] and Arachchi et al. [3] have also noted an RN-UN connection in the arm. Nevertheless, this is the most commonly described connection between nerves originating from the brachial plexus.

Sun et al. [23] noted that variability regarding RN may also depend on age. Some reports indicate that C5, C6, C7 root injuries in newborns cause RN dysfunction while the same injuries in adults do not impair the RN function. The authors hypothesized that the radial nerve had a lower proportion of myelinated nerve fibers from the lower trunk in newborns than in adults. They confirmed this in their study on six adult and six newborn cadavers [23]. We can definitely say that the amount of nervous fibers in the radial nerve from the lower roots of BP increases with age.

Interconnections between nerves and additional branch to muscles can prove an obstacle while determining the extent of damage to components of the brachial plexus. Non-standard innervation of the coracobrachialis longus by the radial nerve does not only provide invaluable information for operators in the area but also for medical professionals determining the extent of BP damage on the neuromuscular function [13]. This type may falsely resemble symptoms of musculocutaneous nerve neuropathy, due to the fact that the coracobrachialis longus is almost always innervated by it. Being aware of variations, we can greatly improve our ability to detect pathologies and save a lot of time [17].

Additional innervation of the coracobrachialis longus or other muscles can also be beneficial because double innervation significantly reduces the risk of complete muscle paralysis [4]. In our case, we noted that CL was innervated only by the radial nerve. This does not reduce the risk of complete paralysis of the muscle, because this innervation still comes from a single nerve. Moreover, in this case, the risk is even increased because radial nerve injuries occur more frequently than musculocutaneous nerve injuries [11].

**Fig. 1:**
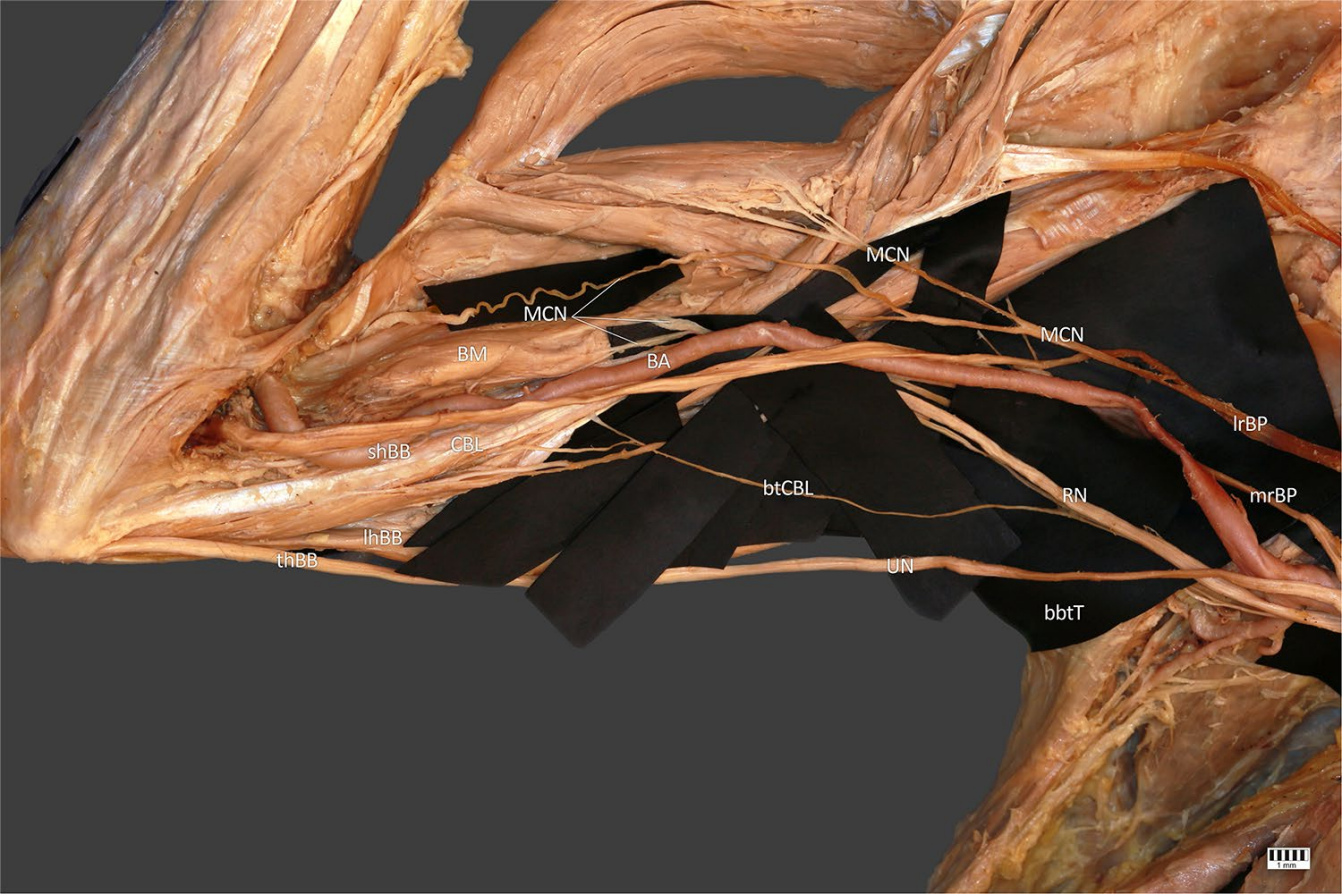
*mrBP* medial root of brachial plexus, *lrBP* lateral root of brachial plexus, *RN* radial nerve, *UN* ulnar nerve, *MC* musculocutaneous nerve, *btCBL* branch to coracobrachialis longus, *CBL* coracobrachialis longus, *BA* brachial artery, *B* brachialis, *btT* branch to triceps, *lhBM* long head of biceps brachii, *shBM* short head of biceps brachii, *tBM* third head of biceps brachii

The anatomical location of the new branch is as important as its biomechanical impact. Due to its high departure point from the radial nerve in its initial course, it may resemble branches to the triceps brachii muscle [21]. It is not easy to distinguish these branches from coracobrachialis branch of RN (CBB) because their standard departure points described by Cho et al. [7] are very close to those we observed in our measurements. From our observation, the close proximity of the superior ulnar collateral artery in the distal part of CBB and its smaller diameter may be helpful. We believe that superior ulnar collateral artery could be considered a landmark for CBB. However, more information about CBB occurrence is needed (Fig. 1).

CL is relatively rare in any population. Therefore, we do not have much information about its innervation. There are reports on CL provided by MCN [9, 18, 32]. Because of that CL is a variant of CBM, we can expand our research for coracobrachialis muscle (CBM). CBM is also mainly innervated by MCN. Moreover, available literature presents individual cases describing innervation of CBM by the median nerve [27, 29]. To our knowledge, the branch from RN to the coracobrachialis muscle was not previously described. This is not surprising because the radial nerve does not normally innervate any muscle from the posterior group of arm, and cases like ours are extremely rare.

**Fig. 2:**
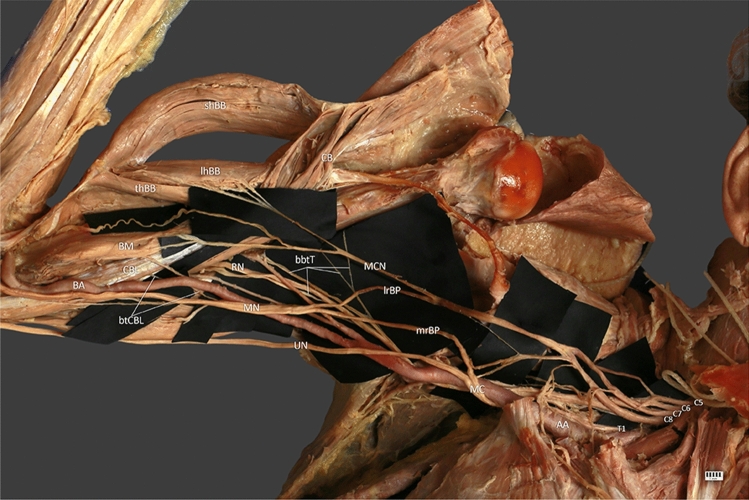
*mrBP* medial root of brachial plexus, *lrBP* lateral root of brachial plexus, *RN* radial nerve, *UN* ulnar nerve, *MC* musculocutaneous nerve, *btCBL* branch to coracobrachialis longus, *CBL* coracobrachialis longus, *BA* brachial artery, *B* brachialis, *btT* branch to triceps, *lhBM* long head of biceps brachii, *shBM* short head of biceps brachii, *tBM* third head of biceps brachii, *MC* medial cord, *MN* median nerve, *bMHT* branches to medial head of triceps, *AA* axillary artery

It is also worth mentioning the co-occurrence of the variability we described above with the 3-headed biceps brachii muscle (Fig. 2). The BB is one of the most variable muscles in the human body [24–26]. The occurrence of additional heads is often associated with variations of the nerve (for example its doubling) and atypical course of arteries [2, 31]. Moreover, Yamamoto et al. [30] show in their study clear correlation between the presence of an additional head of the biceps brachii and penetrate of the BB by the MCN. It is no different in case we observed. The relationship between the origins of the shBB and CBM is also variable [33]. This case represents the most common combination of their origins (54%) and represents type I in the classification presented by Zielinska et al. [33] (one head of the CBM and the shBB creating a common junction originating from the coracoid process).

## Conclusion

Anatomy and muscle supply through the brachial plexus are morphologically variable and known quite well. However, the cases are characterized with hardly any changes and their occurrence can cause many problems regarding diagnostics, surgical procedures, and nerve blocks. Knowledge of the presence of an additional and non-standard branch, especially innervating muscles, could significantly improve the surgical procedure, treatment process and in some cases, enables to make a proper diagnosis.

## Data Availability

Please contact authors for data requests (Łukasz Olewnik PhD—email address: lukasz.olewnik@umed.lodz.pl).
